# DNA Methylation Signatures Triggered by Prenatal Maternal Stress Exposure to a Natural Disaster: Project Ice Storm

**DOI:** 10.1371/journal.pone.0107653

**Published:** 2014-09-19

**Authors:** Lei Cao-Lei, Renaud Massart, Matthew J. Suderman, Ziv Machnes, Guillaume Elgbeili, David P. Laplante, Moshe Szyf, Suzanne King

**Affiliations:** 1 Department of Psychiatry, McGill University and Psychosocial Research Division, Douglas Hospital Research Centre, Montreal, Quebec, Canada; 2 Department of Pharmacology and Therapeutics, McGill University, Montreal, Quebec, Canada; 3 Department of Pharmacology and Therapeutics, Sackler Program for Epigenetics and Developmental Psychobiology and McGill Centre for Bioinformatics, McGill University, Montreal, Quebec, Canada; 4 Psychosocial Research Division, Douglas Hospital Research Centre, Montreal, Quebec, Canada; 5 Department of Pharmacology and Therapeutics and Sackler Program for Epigenetics and Developmental Psychobiology, McGill University, Montreal, Quebec, Canada; University of Tokyo, Japan

## Abstract

**Background:**

Prenatal maternal stress (PNMS) predicts a wide variety of behavioral and physical outcomes in the offspring. Although epigenetic processes may be responsible for PNMS effects, human research is hampered by the lack of experimental methods that parallel controlled animal studies. Disasters, however, provide natural experiments that can provide models of prenatal stress.

**Methods:**

Five months after the 1998 Quebec ice storm we recruited women who had been pregnant during the disaster and assessed their degrees of objective hardship and subjective distress. Thirteen years later, we investigated DNA methylation profiling in T cells obtained from 36 of the children, and compared selected results with those from saliva samples obtained from the same children at age 8.

**Results:**

Prenatal maternal objective hardship was correlated with DNA methylation levels in 1675 CGs affiliated with 957 genes predominantly related to immune function; maternal subjective distress was uncorrelated. DNA methylation changes in *SCG5* and *LTA*, both highly correlated with maternal objective stress, were comparable in T cells, peripheral blood mononuclear cells (PBMCs) and saliva cells.

**Conclusions:**

These data provide first evidence in humans supporting the conclusion that PNMS results in a lasting, broad, and functionally organized DNA methylation signature in several tissues in offspring. By using a natural disaster model, we can infer that the epigenetic effects found in Project Ice Storm are due to objective levels of hardship experienced by the pregnant woman rather than to her level of sustained distress.

## Introduction

Prenatal maternal stress (PNMS) predicts a wide variety of outcomes in the offspring [1]. Testing the ‘fetal programming hypothesis’, animal studies randomly assign pregnant rodents to stress or non-stress conditions and find that maternal glucocorticoids (GCs) pass the placenta and alter fetal brain development [2]. In addition, GCs alter the hypothalamic-pituitary-adrenal (HPA) axis and the immune system in the fetus [3]. Experimental research with non-human primates shows that *in utero* exposure to even mild stressors can produce permanent changes in metabolic, immune and behavioral systems in the fetus [4], [5]. Retrospective epidemiological studies show that severe PNMS in humans, such as that caused by military invasion, increases risk for a variety of disorders in the offspring including schizophrenia [6]. Prospective human studies suggest that maternal anxiety and life events in pregnancy predict the fetus' risk for cognitive and behavioral problems in later life [7].

Epigenetic modification of gene function may be one mechanism by which PNMS results in poor outcomes in the offspring. DNA methylation, an intensively studied epigenetic mechanism, could be modulated by exposure to a variety of maternal experiences and might participate in processes that “adapt” the genome to stress signals across multiple tissues and explain the broad-ranging effects of early life stress on the fetus [8], [9]. Growing evidence from human and animal studies suggests that DNA methylation is involved in effects of PNMS on outcomes in offspring [10]–[16]. For example, in rats, chronic restraint stress in the pregnant dam affects methylation levels and expression of *11βHSD2*, *DNMT3a* and *DNMT1* in placenta and brain [10]. In a different PNMS study using prenatal bystander stress in rats, global DNA methylation was altered in hippocampus and frontal cortex in the offspring [11]. In humans, prenatal exposure to maternal depressed mood was correlated with *SLC6A4* methylation level in infants' cord blood [12]. Moreover, Liu et al. showed that depression in pregnancy was associated with methylation in imprinted genes in cord blood [13]. Furthermore, three independent studies demonstrated that methylation status of *NR3C1* promoter in cord blood was predicted by maternal depressed mood in third trimester [14], by prenatal maternal war-related stress [15] and by partner violence during pregnancy [16].

Thus, both animal and human research suggest that *in utero* exposure to some form of maternal “stress” or mood correlates with the fetus' epigenome. None of these animal or human studies are capable, however, of determining which aspect of the stress experience is responsible for triggering a biological cascade that will reach the fetus to alter development: whether the objective hardship experienced by the pregnant female, or her level of subjective distress, or some combination of the two. Because random assignment to stress and non-stress groups by the researcher is impossible with pregnant humans, there is always the threat to internal validity that the results may be the result of pre-existing genetic or environmental confounders rather than to the effect of the stressor *per se*. What is needed is a human model that approximates the random assignment to stress conditions that is possible when working with laboratory animals, yet is generalizable to the human stress experience. One approach to circumventing these methodological challenges is to study the effects of exposure to an independent random stressor, such as a natural disaster, on DNA methylation, thereby isolating any effects of the mother's objective degree of exposure from any genetic predispositions, and from her subjective level of distress.

Project Ice Storm was conceived following one of Canada's worst natural disasters in history: the January 1998 Quebec ice storm. Between January 6 and January 9, a series of freezing rain storms passed through southern Quebec covering everything in a layer of ice. The weight of the ice toppled high tension power lines and utility poles, collapsing the power grid, particularly in the Montérégie region of Quebec. Resulting power outages ranged from a few hours to as long as 6 weeks for three million people in the province of Quebec. On Friday January 9, the downtown core of Montreal was blacked out, leaving the city in darkness and commuters stranded in metro cars. The military were called in to assist local forces in removing broken trees and other debris from roads. Cold fronts followed the mild weather, plunging the region into seasonal temperatures of −10C to −20C. Without electricity, central heating, pumps for well water, farm and factory equipment stopped working. Security forces went door to door to rescue isolated individuals in danger from cold and hypothermia, asphyxiation from unconventional heating devices, and fire due to blocked chimneys. There were more than 27 deaths attributed to the ice storm. The total insurance payouts were $1.5 billion CAD and an additional $1.5 billion CAD in losses were covered by the government and industry [17]. The personal and financial costs of the disaster left a significant impact on the population. Project Ice Storm has found that maternal objective hardship and subjective distress predict different sets of developmental outcomes [18]. The Project Ice Storm cohort provides a unique opportunity to determine whether a direct relationship exists between *in utero* exposure to maternal stress and DNA methylation signatures in the offspring, and to determine the extent to which variance in methylation is explained by objective and/or subjective PNMS. In this cohort, the mothers' degree of objective hardship is uncorrelated with demographic characteristics such as socioeconomic status, education and income; as well, there is only a low correlation between objective hardship and subjective distress (r<.30)[18].

It is known that DNA methylation patterns are involved in defining cell-specific genome programs. Therefore, it has been assumed that DNA methylation differences related to behavior would be limited to particular brain regions. We hypothesized, however, that since the outcomes that are associated with exposure to PNMS are both physical and behavioral, the DNA methylation changes that mediate such effects should be found in a variety of tissues, even peripheral cells [19]. We focused on the immune system because of the tight bidirectional relationship between the immune system and the brain, particularly the HPA axis, which coordinates the system-wide response to stress [20], [21]. We chose a specific white blood cell (WBC), CD3+ T cells, to reduce confounding cell-type specific differences in DNA methylation between different WBC. We then examined whether several of the DNA methylation differences triggered by PNMS are also present in other tissue sources such as saliva and whole blood cells that are more accessible sources than brain in standard longitudinal behavioral studies in humans.

Thus, the objectives of this study were (a) to determine the extent to which objective and/or subjective PNMS from a natural disaster would explain variance in DNA methylation patterns many years after birth; and (b) to determine whether these patterns can be discerned in T cells, peripheral blood mononucleur cells (PBMCs), and saliva cells.

## Materials and Methods

(Note: A detailed description of all experimental and statistical methods is provided in the online File S1)

### Participants and Measures

Project Ice Storm recruited 176 mothers, who were pregnant during the January 1998 Quebec ice storm or who conceived within 3 months of the storm when stress hormones could still be elevated. All women were living in the Montérégie region southeast of Montreal at the time of the storm, were native French speakers, ethnically Caucasian, and were aged 18 years or older [22]. In June 1998, storm-related PNMS was assessed using two questionnaires. A 32-point questionnaire assessed degree of objective hardship (Storm32) including questions about loss (e.g., damage to residence), scope (e.g., number of days without electricity), threat (e.g., injury to self), and change (e.g., time in a shelter) [23]. To measure subjective distress, women also completed a validated French version [24] of the Impact of Events Scale-Revised (IES-R) [25] which assesses the severity of post-traumatic stress-like symptoms (hyperarousal, avoidance, intrusive thoughts and images) related to the ice storm.

Thirty-six youth from the study (20 males; 16 females) agreed to provide blood samples for epigenetic analyses when they were, on average, 13.3 years of age (SD = 0.3) in 2011. There were 8 youth whose mothers became pregnant after the ice storm occurred, and 28 youth whose mothers were already pregnant at the time of the ice storm; no significant differences were found between these groups in terms of gender, or levels of objective and subjective PNMS. Thirty-four of these thirty-six children had also provided saliva samples at age 8 years (19 males; 15 females). The children's health status and medication use was screened before the blood draw.

### Ethics Statement

After a complete description of the study to the subjects, we obtained written informed consent from parents, and written assent from adolescents. This study was approved by the Research Ethics Board of the Douglas Hospital Research Center.

### T cell isolation and DNA extraction

T cells were isolated from PBMCs by immunomagnetic separation with Dynabeads CD3 (Dynal, Invitrogen). DNA extraction from T cells and PBMCs was performed using Wizard Genomic DNA Purification kit (Promega) according to the manufacturer's instructions.

### Saliva collection and DNA extraction

Saliva was collected using Oragene DNA self-collection kit (OG-500) (DNA Genotek Inc.). DNA extraction was performed using PrepIT-L2P kit (DNA Genotek Inc.) according to the manufacturer's instructions.

### Infinium Human Methylation 450 BeadChip Array

We evaluated the effect of maternal exposure to the disaster on DNA methylation from the T cells of 34 youth; DNA methylation from 2 youth was not obtained due to very low T cell DNA concentrations. We used Illumina Infinium Human Methylation 450 BeadChip Array to determine DNA methylation levels in T cells at 480,000 CGs across the genome and then correlated the levels of methylation with the degree of objective and subjective PNMS. CGs with an inter-quartile range (IQR) less than .10 (i.e., 10% methylation difference) were removed. Furthermore, since samples were obtained from both males and females, CGs for chromosomes X and Y were excluded. The remaining 10,553 probes were tested for association with object hardship (Storm32 score), and subjective distress (IES-R total score). To correct for multiple testing, the Benjamini-Hochberg algorithm was used to compute the false discovery rate (FDR) from the p-values and FDR was set at <0.2 (for detailed statistical method see: ***File S1***). Infinium Human Methylation 450 BeadChip Array analysis was completed by Genome Quebec according to standard protocols.

### Bisulfite treatment and pyrosequencing

Bisulfite treatment of 250 ng genomic DNA was performed using the EZ DNA Methylation-Gold Kit (Zymo Research), and pyrosequencing was performed using PyroMarkQ24 (Qiagen). The primers, PCR amplification conditions, and sequencing protocols for the bisulfite pyrosequencing are shown in ***File S1, Table S1 and S2***.

### *SCG5* promoter cloning

Two fragments of *SCG5* promoter were cloned by PCR amplification from the human embryonic kidney (HEK) cell cDNA at positions 32933343-32933992 and positions 32933343-32934034 (chromosome 15) into the pCpGL-reporter containing the luciferase reporter gene at the *Bgl*II and *Nco*I restriction sites [26].

### *In vitro* pCpGL-SCG5 promoter methylation

Plasmid constructs were methylated *in vitro* using CpG methyltransferase (M.*Sss*I) (New England Biolabs).

### Cell line and transfection

Human embryonic kidney 293 cells (HEK293) (ATCC) were transiently transfected using calcium phosphate method based on Rouleau *et al* [27].

### Luciferase activity assay

The lysates were assayed for luciferase activity 48 h later using Luciferase assay substrate (Promega, USA) and the reactions were read using Lumat LB9507 (Berthold Technologies, Germany).

### Ingenuity pathway analysis (IPA)

Differentially methylated genes were classified by IPA software (www.ingenuity.com). A right-tailed Fisher's exact test was used to calculate the Gene enrichment. Biological functions with a cut off p-value less than 0.05 were considered statistically significant.

### Statistical Analysis

The Illumina Infinium Human Methylation 450 BeadChip Array statistical analyses were performed using R packages. All other analyses were performed using SPSS (Version 20, SPSS Inc., Chicago IL, USA) and associations were calculated using Pearson's correlation coefficient which was corrected according to Bonferroni. All p-values reported are two-sided.

## Results

### Effects of PNMS on genome-wide DNA methylation profiling

**Figure 1:**
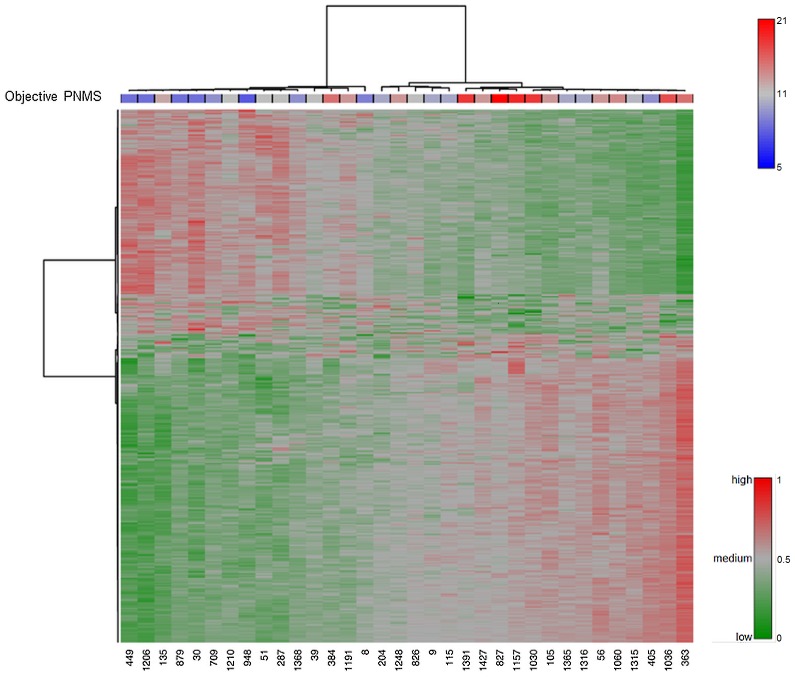
Differentially methylated CGs responding to objective PNMS (Storm32 score). Heatmap represents the DNA methylation levels of 500 CGs most significantly associated with objective PNMS (Storm32 score) in 34 donors. Each column represents an individual and each row a single CG. Each cell represents the CG methylation level for one site in one sample. A color gradient intensity scale at the lower right-hand corner of the Heatmap expresses methylation changes. The darkest green indicates the lowest methylation level (Beta-value = 0), the gray indicates the median score (Beta-value = 0.5) and the darkest red indicates the highest methylation level (Beta-value = 1). The color bar on the top of the Heatmap indicates subjects' categorization by their mother's objective PNMS. A color gradient intensity scale at the higher right-hand corner of the Heatmap shows the level of objective PNMS. The darkest blue indicates the lowest objective PNMS (Storm32 score = 5), the gray indicates the median objective PNMS (Storm32 score = 11) and the darkest red indicates the highest objective PNMS (Storm32 score = 21).

Storm32 scores ranged from 5–21 (Mean = 10.9, SD = 4.2) and IES-R scores ranged from 0–40 (Mean = 9.5, SD = 9.2). Remarkably, no statistically significant correlations were found between subjective PNMS (IES-R) and methylation levels for any CGs. In contrast, the methylation levels of 1675 CGs were significantly correlated with objective PNMS (Storm32) (823 CGs were positively correlated and 852 were negatively correlated) (***Table S3***). The Heatmap of the 500 CGs which were most strongly associated with the degree of objective PNMS is presented in **Fig. 1** and reveals a dose-response relationship with DNA methylation state. Hierarchical cluster analysis of individual methylation patterns was performed; the results are represented in a dendrogram as shown on the top and left of the Heatmap. Significant CGs were identified in 22 chromosomes (probes for chromosomes X and Y were excluded), revealing that objective PNMS triggered a broad signature in the genome. 122 (7.3%) of the CGs were located in CpG island, 119 (7.1%) and 109 (6.5%) were located in N-shelf and S-shelf, 222 (13.3%) and 155 (9.3%) in N-shore and S-shore, respectively, and the rest (56.6%) were located in the open sea. 107 differentially methylated CGs were in immediate proximity (200 bp) of transcription start site (TSS), 206 were 1500 bp away from TSS, 275 were in the 5′UTR and 80 are in the first exon. 749 CGs were in gene bodies and 82 were located in 3′UTR. A total of 957 genes were associated with the 1675 differentially methylated CGs, 281 of these genes had multiple differentially methylated CGs and 677 had significant DNA methylation differences in only one CG contained on the array, while 46 CGs were affiliated with more than one gene. Interestingly, *LTA* (lymphotoxin alpha), which is involved in regulating the innate and adaptive immune system [28], had the most CGs (18 differentiated methylated CGs) that correlated with objective PNMS.

We investigated whether sex moderated the association between objective PNMS and DNA methylation, however, no significant interaction effect was found.

### Validation of correlation between degree of objective PNMS and site-specific CG methylation levels by pyrosequencing

**Figure 2:**
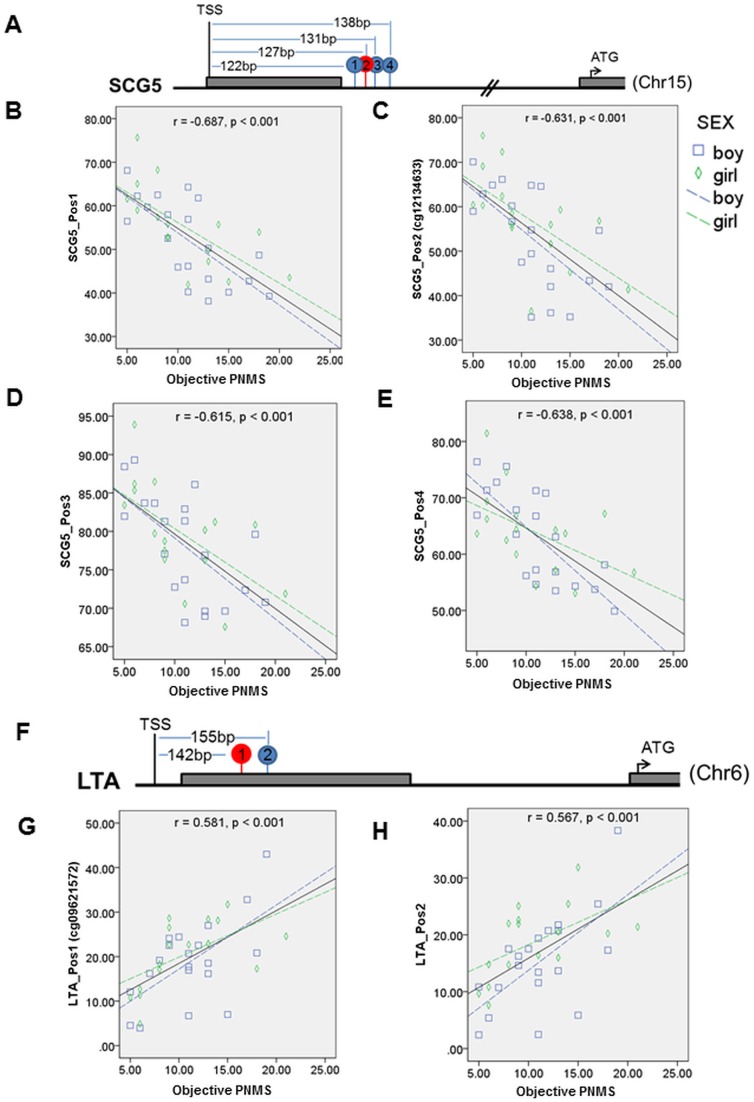
The correlation between objective PNMS and methylation data from pyrosequencing in *SCG5* and *LTA*. A) Physical map of CGs in the *SCG5*. Grey bars represent the exons. CG labeled in red represents the interrogated CG and that labeled in blue represents the immediately surrounding CGs. B–E) Correlations between objective PNMS and methylation level of Positions1, 2(cg12134633), 3 and 4. F) Physical map of CGs in the *LTA*. G–H) Correlations between objective PNMS and methylation level of Position1 (cg09621572) and 2. Blue squares indicate male and green diamonds indicates female. Dashed blue line represents the fitting line in males and green in females.

Validation of the 450 K BeadChip Array DNA methylation data was performed with pyrosequencing of bisulfite-treated DNA in 36 youth. We examined 9 genes containing 12 CGs amongst the top 500 CGs whose level of methylation significantly correlated with degree of objective PNMS (***Table S4***). These genes were selected according to their CG locations, the correlation coefficients, and the gene functions. There was a strong correlation between 450 K BeadChip Array beta-values for each CG and the methylation levels obtained by pyrosequencing (r = 0.931, p<0.001) (***Fig. S1***). Ten out of 12 CGs investigated by pyrosequencing exhibited a significant correlation between their level of methylation and the degree of objective PNMS. For example, the cg12134633 in *SCG5* (Secretogranin V), located 127 bp downstream of transcription start site (**Fig. 2A**), exhibits a high negative correlation with objective PNMS in 450 K BeadChip Array data; consistent with this finding, pyrosequencing revealed a high negative correlation (r = −0.631, p<0.001) not only between methylation level of the cg12134633 included on the array and objective PNMS (**Fig. 2C**) but also in additional surrounding CGs in the same region (**Fig. 2B, D and E**), suggesting that the differential methylation of the CGs included in the 450 K BeadChip Array represents the state of methylation of the entire 5′ region. Likewise, our analysis shows a high positive correlation (r = 0.581, p<0.001) between methylation level of cg09621572 in *LTA* and objective PNMS (**Fig. 2F and G**) which was consistent in another CG in this region (r = 0.567, p<0.001) (**Fig. 2H**). Similar results were found for other CGs (***Fig. S2***).

### Gene Pathways involved in the immune system are prominently affected by changes in DNA methylation in response to objective PNMS

**Figure 3:**
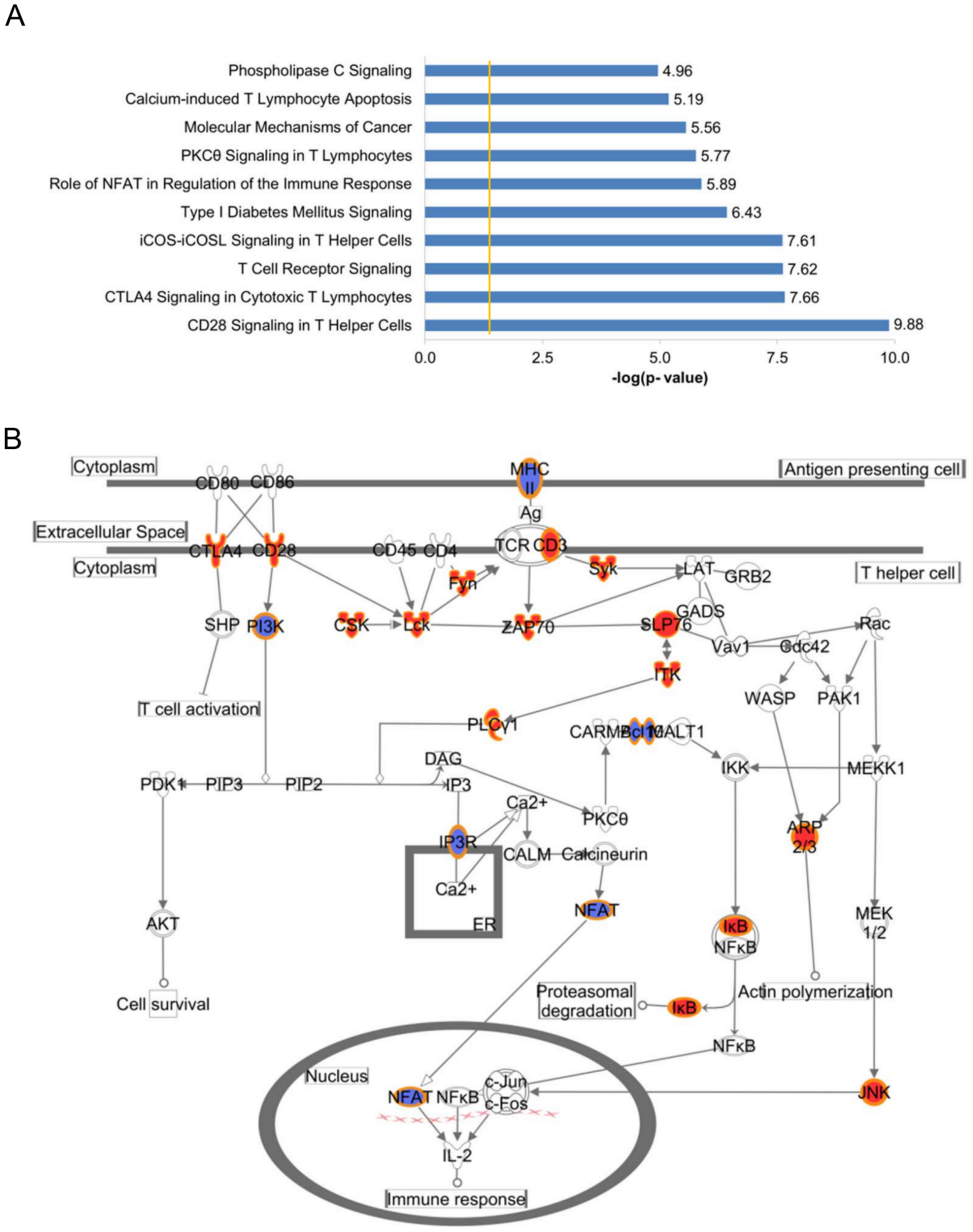
The molecular and cellular functions of the 957 genes analyzed with IPA. A) Top 10 functions of the 957 differentially methylated genes. The y-axis shows functions while the x-axis shows -log(p-value). The yellow line indicates the threshold value of p<0.05. B) The most significant canonical pathway: CD28 Signaling in T helper cells. Genes whose methylation levels are positively correlated with objective PNMS are colored in red and those whose methylation levels are negatively correlated with objective PNMS are colored in blue. CD247: CD247 molecule; FYN: a membrane-associated tyrosine kinase; CD3E: CD3-epsilon polypeptide; CSK: C-Src Tyrosine Kinase; PLCG1: Phospholipase C, Gamma 1; NFATC1: Nuclear Factor Of Activated T-Cells, Cytoplasmic, Calcineurin-Dependent 1; HLA-DMB: Major Histocompatibility Complex, Class II, DM Beta; ITPR1: inositol 1,4,5-trisphosphate receptor, type 1; CD3D: CD3d Molecule, Delta; CTLA4: cytotoxic T-lymphocyte-associated protein 4; CD3G: CD3-gamma polypeptide; CD28: CD28 Molecule; LCK: lymphocyte-specific protein tyrosine kinase; ACTR3: ARP3 Actin-Related Protein 3 Homolog (Yeast); NFKBIA: nuclear factor of kappa light polypeptide gene enhancer in B-cells inhibitor, alpha; BCL10: B-Cell CLL/Lymphoma 10; SYK: spleen tyrosine kinase; ZAP70: zeta-chain (TCR) associated protein kinase 70 kDa; ARPC4: actin related protein 2/3 complex, subunit 4; MAPK10: mitogen-activated protein kinase 10; HLA-DOB: Major Histocompatibility Complex, Class II, DO Beta; PIK3CD: phosphatidylinositol-4,5-bisphosphate 3-kinase, catalytic subunit delta; PIK3R2: phosphoinositide-3-kinase, regulatory subunit 2 (beta); LCP2: Lymphocyte Cytosolic Protein 2; ITK: IL2-inducible T-cell kinase.

A total of 957 genes were examined to determine whether they are significantly related to any biological functions or diseases according to the Ingenuity Pathway Analysis (IPA) database (www.ingenuity.com) (a detailed summary of the pathway analysis is presented in ***Table S5***.). **Fig. 3A** charts the top 10 canonical pathways. Interestingly, pathways involved in immune system are prominent: the top pathway is CD28 signaling in T Helper cells; 25 of the 132 genes included in this pathway were found to be correlated with objective PNMS in the present study (**Fig. 3B**) (p = 1.32E10^−10^). Except for *HLA-DMB*, *Bcl10*, *HLA-DOB, NFATC1* and *PIK3R2*, the rest of the 20 genes in the pathway were hyper-methylated with increased levels of objective PNMS. CD28 is a co-receptor for the TCR/CD3 complex and is responsible for providing the co-stimulatory signal required for T cell activation [29]. Nineteen genes from the current study were involved in CTLA4 signaling in Cytotoxic T lymphocytes pathway (p = 2.2E10^−8^) (***Fig. S3***) in which CTLA-4 plays a role in down-regulating T cell responses [29], [30]. Together, 44 genes from our study are involved in “turning on” and/or “turning off” T cell activation, suggesting that objective PNMS may have an important effect on immune function which is consistent with the immune phenotype that is usually associated with early life stress. Furthermore, highly significant enrichments in biological functions have been observed to be related to the immune system; for example, Inflammatory Response (p<1.15E10^−16^–2.06E10^−4^), Immunological Disease (p<1.11E10^−11^–1.98E10^−4^), Hematopoiesis (p<7.52E10^−28^–2.91E10^−4^) and Cell-mediated Immune Response (p<5.87E10^−27^–2.62E10^−4^) were frequently encountered. Moreover, the potential upstream regulators of the differentially methylated genes such as TCR (p = 4.34E10^−12^), IL15 (p = 2.25E10^−10^), CD3 (p = 4.48E10^−10^), TNF (p = 2.68E10^−8^) and dexamethasone (p = 2.20E10^−5^) have been observed. Although the biological functions of the significantly differentiated genes in the present study were predominantly involved in immune system, genes involved in metabolic functioning were also affected by objective PNMS. For example, the methylation patterns of 19 of the 120 genes involved in the Type I diabetes Mellitus signaling pathway (p = 3.73E10^−7^) were significantly correlated with objective PNMS levels (***Fig. S4***).

### DNA methylation states that correlate with objective PNMS are detectable in PBMCs- and saliva-derived DNA

One of the main challenges in behavioural epigenetics is the fact that the brain is inaccessible to epigenetic research in living humans. Because DNA methylation patterns exhibit high tissue specificity [31], it is not anticipated that brain specific genes will exhibit change in DNA methylation in the periphery. Therefore, in this study we focused on a peripheral, physiologically-relevant tissue for stress: the immune system. As expected, immune-related genes were highly affected by objective PNMS, however, the feasibility of using extracted T cells is limited in many psychosocial studies. Therefore, we used pyrosequencing to determine whether the DNA methylation signatures of PNMS that we had identified in T cells could be observed in PBMCs as well as in biological samples that are commonly collected in psychosocial and public health studies: saliva. We examined the correlations between *SCG5* and *LTA* CGs methylation levels and objective and subjective PNMS in PBMCs and saliva samples obtained from the same subjects. The methylation levels of 4 CGs in *SCG5* and 2 CGs in *LTA* were significantly and highly correlated with objective PNMS in PBMCs and saliva samples in this cohort (**Table 1**). As was observed in isolated T cells, there were no statistically significant correlations between subjective PNMS and DNA methylation levels in *SCG5* and *LTA* in PBMCs or saliva samples (data not shown). As expected, we found highly significant correlation of DNA methylation patterns between T cells, PBMCs and saliva DNA (**Table 1**).

**Table 1:** Correlations between objective PNMS and methylation levels of CGs in *SCG5* and *LTA* in 3 cell types.

Cell types	SCG5				LTA	
	Pos 1	Pos 2 (cg12134633)	Pos 3	Pos 4	Pos 1 (cg09621572)	Pos 2
T cells	−.687**	−.631**	−.615**	−.638**	.581**	.567**
PBMCs	−.551**	−.516**	−.524**	−.567**	.489**	.706**
Saliva	−.602**	−.434*	−.428*	−.512**	.484**	.387*
T cells vs PBMCs	.552**	.482**	.411*	.552**	.512**	.436**
T cells vs Saliva	.498**	.431*	.599**	.581**	.626**	.655**
PBMCs vs Saliva	.500**	.576**	.459**	.618**	.649**	.418*

**. Correlation is significant at the 0.01 level (2-tailed); *. Correlation is significant at the 0.05 level (2-tailed).

Taken together, our observations suggest that informative DNA methylation changes are triggered by objective PNMS, but not by subjective PNMS in pregnancy, at least not in the context of a natural disaster. As well, these effects are detectable not only in T cells but also in PBMCs- and saliva-derived DNA which is methodologically important for following up these DNA methylation signatures in larger studies or with younger children where saliva DNA might be the only source.

### Functional effects of *SCG5* promoter hypermethylation

**Figure 4:**
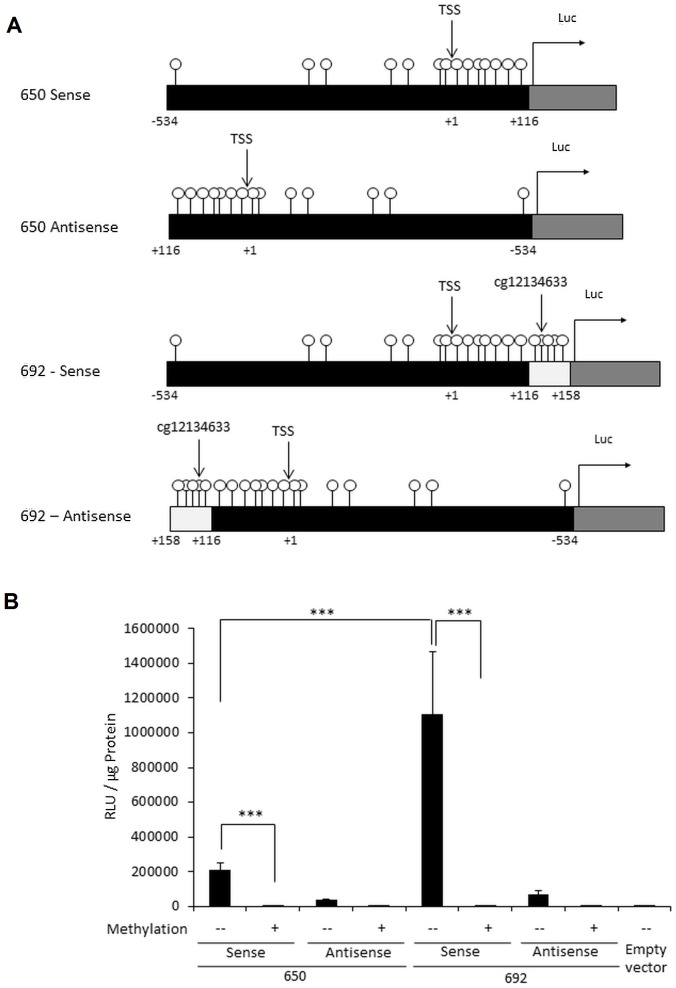
The effect of DNA methylation on *SCG5* promoter activity. A) Schematic representation of the location of CGs investigated in the *SCG5* promoter. The CGs are denoted as lollipops and the +1 position indicates the transcription start site (TSS). White bar indicates the region that contains the 4 differentially methylated CGs. Gray bar indicates the luciferase reporter gene in pCpGL-reporter. The two fragments of 650 bp and 692 bp from the SCG5 promoter region were cloned into the *Bgl*II and *Nco*I restriction sites in pCpGL-reporter in sense and anti-sense orientation, respectively. B) Relative luciferase activity of two promoters region before and after mock methylation (−) or complete in vitro methylation with CpG methyltransferase (M.*Sss*I) (+) and transient transfection (48 h) into in HEK293 cell line (***P<0.001). Promoter activity was normalized to protein concentration. The values are the averages of at least three independent experiments. Data are mean ± SEM.

The greatest effect of PNMS on DNA methylation was found in *SCG5*: higher objective PNMS was associated with lower DNA methylation. Human *SCG5* (also referred to as secretory granule neuroendocrine protein 1 (*Sgne1*)) [32] is located on chromosome 15 and consists of 6 exons in which exon 1 specifies the 5′UTR of mRNA [33]. *SCG5* is widely expressed in neuroendocrine tissues and the protein functions as a chaperone protein for the proprotein convertase PC2 [33]. The CGs that were differentially methylated by objective PNMS in our study are positioned downstream to the transcription start site (**Fig. 4A**). We tested whether methylation of these CGs would affect the ability of *SCG5* promoter to direct transcription and expression of firefly luciferase enzyme in the reporter. Two constructs of the *SCG5* promoter were generated in the CG-less pCpGL-reporter, allowing exclusive methylation of the inserted *SCG5* regions *in vitro* by the bacterial CG methyltransferase (M.*Sss*I): a 692 bp promoter region that included the region containing the differentially methylated CGs (**Fig. 4A**) and a separate construct that doesn't include this region. The differentially methylated 42 bp region enhances transcription from the promoter of *SCG5* in the unmethylated state (comparison of luciferase activity in the 650 bp versus the 692 bp construct; p<0.001 in **Fig. 4B**). *In vitro* methylation of *SCG5* regulatory region with the bacterial CpG Methyltransferase (M.*Sss*I) significantly decreased *SCG5* promoter activity compared with the unmethylated promoter in construct with 650 bp (p<0.001) and 692 bp (p<0.001) respectively. This suggests that the regulatory regions of *SCG5* are sensitive to methylation.

## Discussion

Disentangling the effects of an external stressor, the mother's subjective distress reactions, her trait levels of mood, the intrauterine environment, and genetic predispositions are extremely difficult in most PNMS study designs. Therefore, in human PNMS research we need to find a model which could allow us to isolate specific elements of the human stress experience. The 1998 Quebec Ice Storm offered a unique opportunity to isolate objective and subjective aspects of PNMS and their associations with offspring phenotypes given that the objective degree of ice storm exposure was quasi-randomly distributed in the population; as such, the objective PNMS was not confounded by genetic, psychological, or socioeconomic stratification. The use of an acute-onset, independent, randomly distributed natural disaster as the prenatal stressor mimics the experimental control inherent in animal research. While studies of rodents enable total experimental control of PNMS, they are unable to tease apart the relative effects of the objective degree of hardship exposure to the pregnant dam from her subjective distress levels. This distinction is important for the human stress experience [34]. To the best of our knowledge this is the first human study investigating the effect of both objective and subjective PNMS from an independent stressor such as a natural disaster on genome-wide DNA methylation levels.

Particular brain regions are obvious candidates for DNA methylation changes in response to psychosocial stress, and this has been demonstrated in animal research [35]–[38] and human post-mortem studies [39]–[41]. Our hypothesis was that the response in DNA methylation states to early stress would be “system wide” [19]; this is because multiple phenotypes have been associated with early life stress including behavioural and psychiatric outcomes as well as immune and metabolic function. We also reasoned that this response would be unique for each cell-type reflecting the particular adaptation of the tissue to the stress response. In our study, in order to minimize the heterogeneity of cell populations, we isolated and analysed the methylation levels in CD3+ T cells which are responsive to stress [42] and to HPA axis functioning [43]. Using genome-wide DNA methylation analyses, we observed that the degree of objective PNMS levels from the ice storm was significantly correlated with the methylation of 1675 CGs; surprisingly and interestingly, no correlations were found with subjective PNMS. Although we have shown that subjective PNMS from the ice storm predicts many behavioral outcomes such as anxiety, depression, and aggression in the children [18], objective PNMS in Project Ice Storm has been shown to be more important than the mothers' subjective distress levels in predicting cognitive outcomes such as IQ and language throughout childhood [22], [44], physical outcomes such as obesity at age 5½ [45], and insulin secretion at age 13 [46]. In the current study, maternal anxiety and depression at the child's age of 13½ years were not associated with objective PNMS (data not shown), suggesting that the effect of objective stress on DNA methylation is not the result of mediation via changes in maternal mood and anxiety. Beyond the sheer magnitude of the epigenetic effects of objective PNMS shown here, both in terms of the number of genes involved and the range of difference in methylation, the fact that these effects can be detected 13 years after birth is most impressive. Similarly, prenatal exposure to famine was associated with a persistent decrease in DNA methylation of the imprinted *IGF2* 60 years later in humans [47]. Thus, we may hypothesize that the effects of objective PNMS on child outcomes may be mediated by these DNA methylation changes which could persist throughout life.

As hypothesized, the changes in DNA methylation in T cells were not limited to candidate genes but involved several important functional gene networks as revealed by IPA analysis (***Fig. 3*** ***, Fig. S3–S4 and Table S5***). Moreover, the response in T cells is not just a “surrogate” of epigenetic changes in the brain but reflects the unique biology of T cells as several of the differentially methylated genes are involved in T cell activation pathways such as CD28 signalling in T Helper cells and CTLA4 signalling in Cytotoxic T lymphocytes. This is consistent with a change in gene programming of the immune system itself in response to stress. Thus, the methylome of the immune system could serve as an important target tissue for studying behavioural and psychosocial epigenetics.

The issue of whether it is possible to study the long term consequences of psychosocial stress without having access to brain tissue is obviously critical for progress in the field. Our data support the idea that the methylome of T cells in stress should be studied within its physiological context and not as a “proxy” for events in the hippocampus or other brain regions. A growing volume of evidence from human [48], [49] rodent [50] and nonhuman primate studies [51] shows that immune function could be affected by PNMS. A number of studies from our group and others have revealed that early-life stress is associated with DNA methylation changes in white blood cells in humans [28], [52]–[55] and in T cells in nonhuman primates [37], with genes involved in immune responses particularly affected. In addition, we also observed genes involved in Type I diabetes Mellitus signalling pathway. This finding is consistent with data from the Project Ice Storm cohort, showing that higher levels of objective PNMS were associated with greater insulin secretion [46].

In order to validate our T cell results using a different approach and on different cell-types, we used another subpopulation of blood cells (PBMCs) and saliva cells and performed pyrosequencing on two candidate genes: *SCG5* and *LTA*. We chose *SCG5* because it possesses the top, most highly correlated CG, and *LTA* because it has the most CGs that correlated with objective PNMS. We show here that objective PNMS had similar effects on DNA methylation of *SCG5* and *LTA* in T cells, PBMCs, and saliva. Thus, using saliva DNA for methylation studies holds great promise for the further delineation and application of DNA methylation signatures of psychosocial exposures, especially since obtaining T cells is rarely feasible in large longitudinal psychosocial studies, particularly when following up young children. However, due to the heterogeneity of cell populations such as buccal epithelial cells, granulocytes and lymphocytes in saliva, we cannot exclude the influence of the T cell methylation changes on saliva DNA. In Project Ice Storm, we were able to collect saliva at earlier ages (age 8) than blood (age 13), which allowed us to elucidate the stability of these differential DNA methylation states. The DNA methylation pattern in saliva samples that were collected when the children were 8 years of age were highly correlated with the DNA methylation pattern in T cells samples obtained when the children were 13 years old (**Table 1**). The results presented here suggest that persistent differential methylation changes responding to objective PNMS were conserved not only at different ages (8 and 13 years) but also in different tissue sources (saliva and blood).

Although this pilot study provides the first evidence that randomly assigned PNMS triggers DNA methylation changes in T cells in humans, future studies with larger sample sizes are warranted to further establish the cause and effect relationship between PNMS and DNA methylation. Due to the low starting material, we were not able to obtain RNA; therefore, the relationship between DNA methylation and steady state mRNA levels in CD3+ T cells needs to be carefully examined in further studies where it will be possible to obtain sufficient biological material. Moreover, our results call for a more careful examination of the interactions between DNA methylation changes in response to stress and health outcomes. Potential confounding variables such as infant stress status need to be taken into account in further studies. Our data included DNA methylation measured in 8 (saliva) and 13 (blood) year old children but did not address the question of whether these DNA methylation signatures emerged at birth or later in response to downstream postnatal stress. Unfortunately, no biological material was collected from the children of the Project Ice Storm cohort at birth. This should hopefully be addressed by future studies of this kind.

In conclusion, we provide data supporting an association between PNMS and genome-wide DNA methylation in the periphery in humans. By using a natural disaster, this model allows us to isolate the degree of objective exposure of the mother to the ice storm with less danger of potential confounding by family psychosocial characteristics, and allows us to make tentative conclusions that the associations we uncovered are causal in nature.

## Supporting Information

Figure S1 **The correlation between objective hardship score (Storm32) and methylation data from Illumina Human Methylation 450 K BeadChip Array in 12 CGs associated with 9 genes.** X-axis indicates the percentage methylation of CGs from pyrosequencing and y-axis indicates the beta-value from 450 K BeadChip. Blue squares indicate male and green diamonds indicates female. Dashed blue line represents the fitting line in males and green in females.
(TIF)

Figure S2 **The correlation between objective hardship score (Storm32) and methylation data from pyrosequencing.** Correlations between objective hardship score (Storm32) and methylation level of CG(s) in (A)*MFSD1*, (B)*CD3G*, (C)*UBASH3A*, (D)*IL24*, (E)*EPHB3*, (F)*ITPKB* and (G)*CD8B*. Blue squares indicate male and green diamonds indicates female. Dashed blue line represents the fitting line in males and green in females. Track on the screenshot of Integrative Genomics Viewer (IGV) window marks the location of the CGs examined using pyrosequencing.
(TIF)

Figure S3 **CTLA4 Signaling in Cytotoxic T Lymphocytes.** Genes whose methylation levels are positively correlated with objective hardship are colored in red and those whose methylation levels are negatively correlated with objective hardship are colored in blue. CD247: CD247 molecule; FYN: a membrane-associated tyrosine kinase; CD3E: CD3-epsilon polypeptide; HLA-DMB: Major Histocompatibility Complex, Class II, DM Beta; CD3D: CD3d Molecule, Delta; CTLA4: cytotoxic T-lymphocyte-associated protein 4; CD3G: CD3-gamma polypeptide; CD28: CD28 Molecule; LCK: lymphocyte-specific protein tyrosine kinase; SYK: spleen tyrosine kinase; ZAP70: zeta-chain (TCR) associated protein kinase 70 kDa; HLA-DOB: Major Histocompatibility Complex, Class II, DO Beta; PIK3CD: phosphatidylinositol-4,5-bisphosphate 3-kinase, catalytic subunit delta; PIK3R2: phosphoinositide-3-kinase, regulatory subunit 2 (beta); LCP2: Lymphocyte Cytosolic Protein 2; PPP2R5C: protein phosphatase 2, regulatory subunit B', gamma; PPP2R5E: protein phosphatase 2, regulatory subunit B', epsilon isoform.
(TIF)

Figure S4 **T Cell Receptor Signaling.** Genes whose methylation levels are positively correlated with objective hardship are colored in red and those whose methylation levels are negatively correlated with objective hardship are colored in blue. CD247: CD247 molecule; FYN: a membrane-associated tyrosine kinase; CD3E: CD3-epsilon polypeptide; CSK: C-Src Tyrosine Kinase; PLCG1: Phospholipase C, Gamma 1; NFATC1: Nuclear Factor Of Activated T-Cells, Cytoplasmic, Calcineurin-Dependent 1; HLA-DMB: Major Histocompatibility Complex, Class II, DM Beta; CD3D: CD3d Molecule, Delta; CTLA4: cytotoxic T-lymphocyte-associated protein 4; CD8B: CD8b molecule; CD3G: CD3-gamma polypeptide; CD28: CD28 Molecule; LCK: lymphocyte-specific protein tyrosine kinase; ACTR3: ARP3 Actin-Related Protein 3 Homolog (Yeast); NFKBIA: nuclear factor of kappa light polypeptide gene enhancer in B-cells inhibitor, alpha; BCL10: B-Cell CLL/Lymphoma 10; ZAP70: zeta-chain (TCR) associated protein kinase 70 kDa; PIK3CD: phosphatidylinositol-4,5-bisphosphate 3-kinase, catalytic subunit delta; PIK3R2: phosphoinositide-3-kinase, regulatory subunit 2 (beta); LCP2: Lymphocyte Cytosolic Protein 2; ITK: IL2-inducible T-cell kinase. PAG1: phosphoprotein associated with glycosphingolipid microdomains 1.
(TIF)

Table S1 **The numbers of CGs and analyzed sequences using pyrosequencing.**
(DOCX)

Table S2 **Forward, reverse, pyrosequencing primer sequences and PCR conditions used for bisulphite sequencing.**
(DOCX)

Table S3 **1675 CGs significantly correlated with objective hardship levels (Storm32).**
(XLSX)

Table S4 **Selected CGs/genes for pyrosequencing.**
(XLSX)

Table S5 **Pathway information.**
(XLSX)

File S1 **Supporting data.**
(DOCX)
