# Supplementary figures and images for: DNA Methylation Signatures Triggered by Prenatal Maternal Stress Exposure to a Natural Disaster: Project Ice Storm
Source: PLoS One. 2014 Sep 19;9(9):e107653. doi: 10.1371/journal.pone.0107653 (PMC4169571; doi:10.1371/journal.pone.0107653)

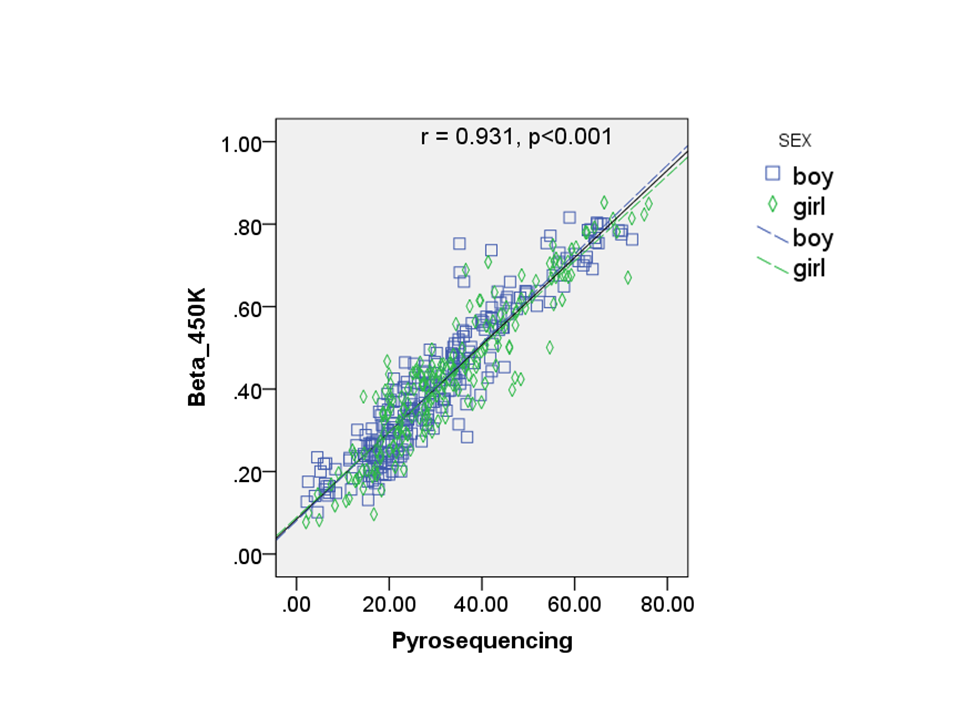

Supplement: Figure S1 — The correlation between objective hardship score (Storm32) and methylation data from Illumina Human Methylation 450 K BeadChip Array in 12 CGs associated with 9 genes. X-axis indicates the percentage methylation of CGs from pyrosequencing and y-axis indicates the beta-value from 450 K BeadChip. Blue squares indicate male and green diamonds indicates female. Dashed blue line represents the fitting line in males and green in females. (TIF) [file pone.0107653.s001.tif]

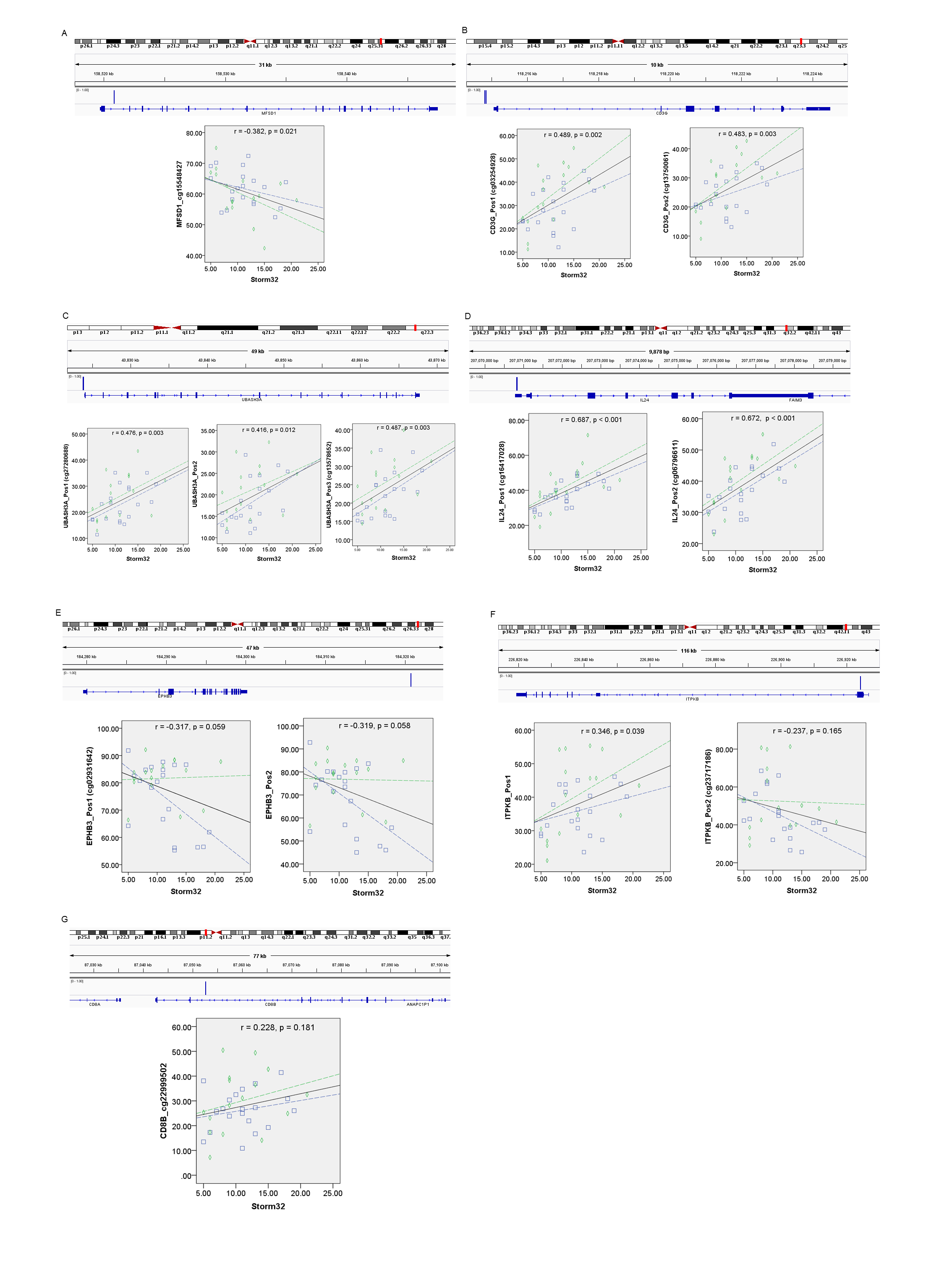

Supplement: Figure S2 — The correlation between objective hardship score (Storm32) and methylation data from pyrosequencing. Correlations between objective hardship score (Storm32) and methylation level of CG(s) in (A)MFSD1, (B)CD3G, (C)UBASH3A, (D)IL24, (E)EPHB3, (F)ITPKB and (G)CD8B. Blue squares indicate male and green diamonds indicates female. Dashed blue line represents the fitting line in males and green in females. Track on the screenshot of Integrative Genomics Viewer (IGV) window marks the location of the CGs examined using pyrosequencing. (TIF) [file pone.0107653.s002.tif]

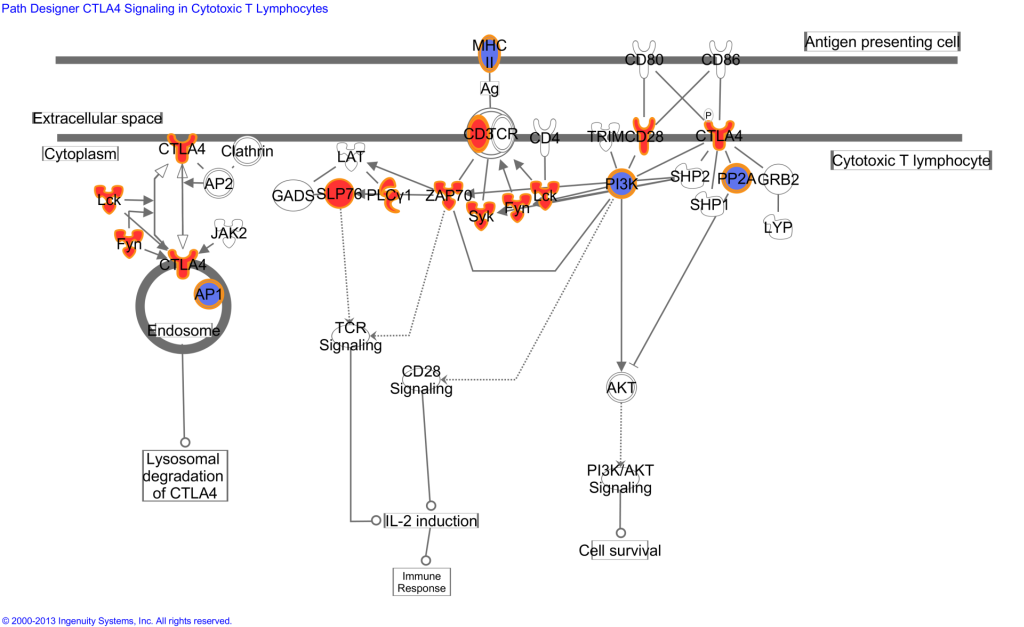

Supplement: Figure S3 — CTLA4 Signaling in Cytotoxic T Lymphocytes. Genes whose methylation levels are positively correlated with objective hardship are colored in red and those whose methylation levels are negatively correlated with objective hardship are colored in blue. CD247: CD247 molecule; FYN: a membrane-associated tyrosine kinase; CD3E: CD3-epsilon polypeptide; HLA-DMB: Major Histocompatibility Complex, Class II, DM Beta; CD3D: CD3d Molecule, Delta; CTLA4: cytotoxic T-lymphocyte-associated protein 4; CD3G: CD3-gamma polypeptide; CD28: CD28 Molecule; LCK: lymphocyte-specific protein tyrosine kinase; SYK: spleen tyrosine kinase; ZAP70: zeta-chain (TCR) associated protein kinase 70 kDa; HLA-DOB: Major Histocompatibility Complex, Class II, DO Beta; PIK3CD: phosphatidylinositol-4,5-bisphosphate 3-kinase, catalytic subunit delta; PIK3R2: phosphoinositide-3-kinase, regulatory subunit 2 (beta); LCP2: Lymphocyte Cytosolic Protein 2; PPP2R5C: protein phosphatase 2, regulatory subunit B', gamma; PPP2R5E: protein phosphatase 2, regulatory subunit B', epsilon isoform. (TIF) [file pone.0107653.s003.tif]

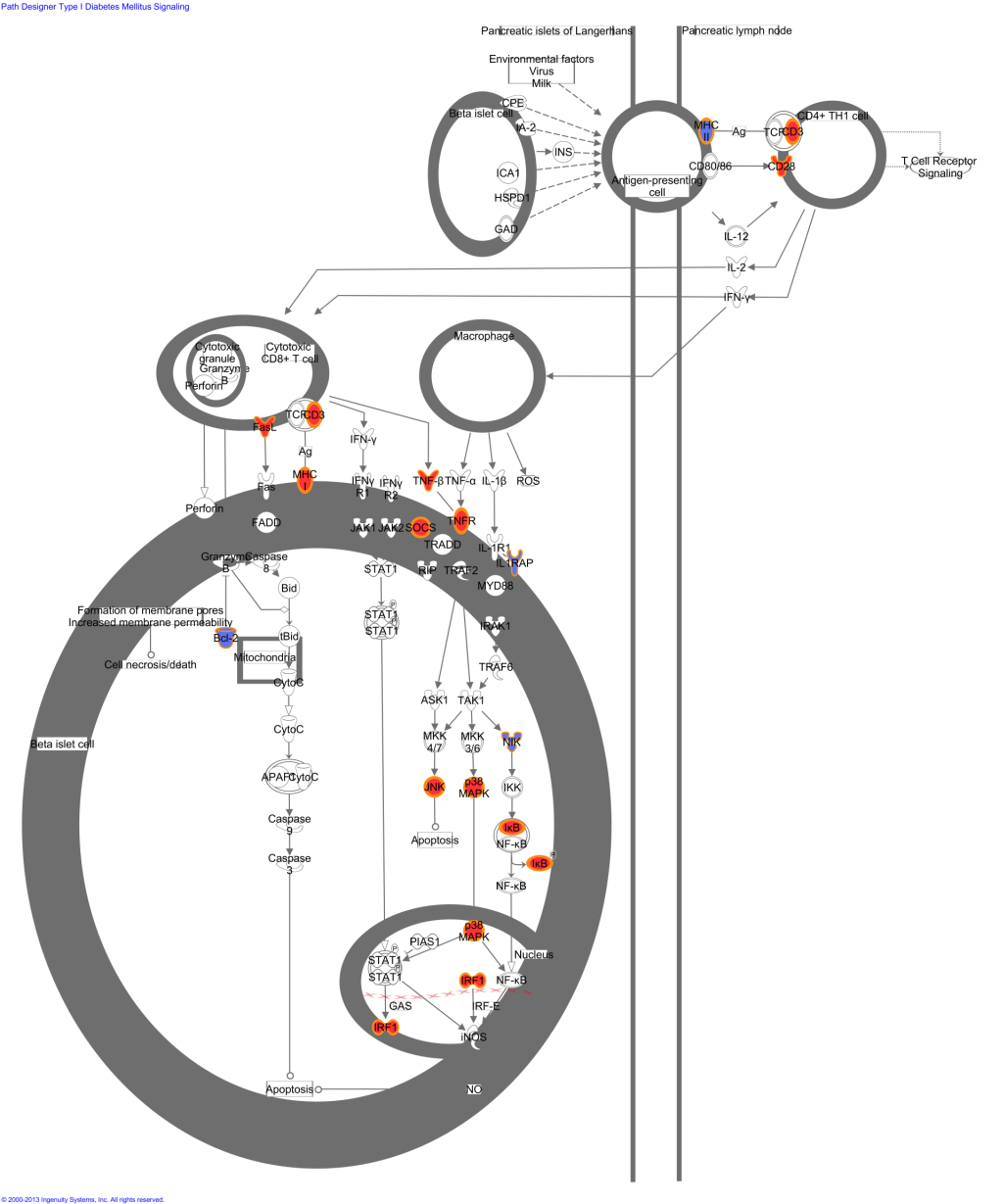

Supplement: Figure S4 — T Cell Receptor Signaling. Genes whose methylation levels are positively correlated with objective hardship are colored in red and those whose methylation levels are negatively correlated with objective hardship are colored in blue. CD247: CD247 molecule; FYN: a membrane-associated tyrosine kinase; CD3E: CD3-epsilon polypeptide; CSK: C-Src Tyrosine Kinase; PLCG1: Phospholipase C, Gamma 1; NFATC1: Nuclear Factor Of Activated T-Cells, Cytoplasmic, Calcineurin-Dependent 1; HLA-DMB: Major Histocompatibility Complex, Class II, DM Beta; CD3D: CD3d Molecule, Delta; CTLA4: cytotoxic T-lymphocyte-associated protein 4; CD8B: CD8b molecule; CD3G: CD3-gamma polypeptide; CD28: CD28 Molecule; LCK: lymphocyte-specific protein tyrosine kinase; ACTR3: ARP3 Actin-Related Protein 3 Homolog (Yeast); NFKBIA: nuclear factor of kappa light polypeptide gene enhancer in B-cells inhibitor, alpha; BCL10: B-Cell CLL/Lymphoma 10; ZAP70: zeta-chain (TCR) associated protein kinase 70 kDa; PIK3CD: phosphatidylinositol-4,5-bisphosphate 3-kinase, catalytic subunit delta; PIK3R2: phosphoinositide-3-kinase, regulatory subunit 2 (beta); LCP2: Lymphocyte Cytosolic Protein 2; ITK: IL2-inducible T-cell kinase. PAG1: phosphoprotein associated with glycosphingolipid microdomains 1. (TIF) [file pone.0107653.s004.tif]
