# Supplementary material for: DNA Methylation Signatures Triggered by Prenatal Maternal Stress Exposure to a Natural Disaster: Project Ice Storm
Source: PLoS One. 2014 Sep 19;9(9):e107653. doi: 10.1371/journal.pone.0107653 (PMC4169571; doi:10.1371/journal.pone.0107653)
Supplement: Table S1 — The numbers of CGs and analyzed sequences using pyrosequencing. (DOCX) [file pone.0107653.s005.docx]

Table S1: The numbers of CGs and analyzed sequences using pyrosequencing.

| **Gene** | **Target ID** | **Illumina Probe ID A** | **Illumina Probe ID B** | **Analyzed sequence using Pyrosequencing (CGs covered)** |
| --- | --- | --- | --- | --- |
| SCG5 | cg12134633 | 23705505 | 39701364 | TYGGTTYGGTYGTTTGTYG (4) |
| MFSD1 | cg15548427 | 70740306 | 70740306 | ATTGGAYG (1) |
| LTA | cg09621572 | 13734408 | 49645473 | TTTTTYGGGTTTTAGTTTYGATTTAGAATTYGTTYGTTGTTTGTTAYGTTGTTATTGTYG (6) |
| UBASH3A | cg27280688 | 48759499 | 48759499 | YGGTTGGATTTTTAGTTTTAAATTTTYGTGGTGGAAATAGTTAGGATTGGTGGAYG (3) |
| UBASH3A | cg13578652 | 44692306 | 44692306 |  |
| CD3G | cg03254928 | 13603502 | 13603502 | AAYGGTTTTAGGATTATTTTTTATTTAGTATTTATTGYG (2) |
| CD3G | cg13750061 | 21635486 | 21635486 |  |
| IL24 | cg16417028 | 26645354 | 26645354 | TTTTAYGGTTGGGAYG (2) |
| IL24 | cg06796611 | 61795504 | 61795504 |  |
| EPHB3 | cg02931642 | 49631320 | 49631320 | TTYGGATTAAGTATTTTTTTTYG (2) |
| ITPKB | cg23717186 | 52644468 | 52644468 | TTYGAGTTTTYG (2) |
| CD8B | cg22999502 | 26639341 | 26639341 | GGTYG (1) |
