# Supplementary material for: DNA Methylation Signatures Triggered by Prenatal Maternal Stress Exposure to a Natural Disaster: Project Ice Storm
Source: PLoS One. 2014 Sep 19;9(9):e107653. doi: 10.1371/journal.pone.0107653 (PMC4169571; doi:10.1371/journal.pone.0107653)
Supplement: Table S2 — Forward, reverse, pyrosequencing primer sequences and PCR conditions used for bisulphite sequencing. (DOCX) [file pone.0107653.s006.docx]

Table S2: Forward (Fwd), reverse (Rev), pyrosequencing (S) primer sequences and PCR conditions used for bisulphite sequencing.

| Genes | Primer sequences | Amplicon size (bp) | Annealing T (**°C**) | [Mg2+](mM) | [Primers] (uM) | PCR cycles |
| --- | --- | --- | --- | --- | --- | --- |
| SCG5_1^st^ round | Fwd: 5’-GGGTTGTTTTTAGGTGAGTATAGTTTTGAT-3’ | 184 | 56 | 2.5 | 0.4 | 40 |
|  | Rev: 5’-CTCAATACTCCCTTCCCCTTAC-3’ |  |  |  |  |  |
| SCG5_nested | Fwd: 5’-GGGTTGTTTTTAGGTGAGTATAGTTTTGAT-3’ | 116 | 56 | 2.5 | 0.4 | 40 |
|  | Rev: 5’-biotin-AACCTCCACCTCAAAAATTTTAACA-3’ |  |  |  |  |  |
| SCG5_S | Fwd: 5’- GGTGAGTATAGTTTTGATG-3’ |  |  |  |  |  |
| MFSD1_1^st^ round | Fwd: 5’-AGATATAGGGTTTTATAATGTTGTTTAGTT-3’ | 230 | 56 | 2.5 | 0.4 | 40 |
|  | Rev: 5’-CAAATTTCATAAAAAAAACCAACCACT-3’ |  |  |  |  |  |
| MFSD1_nested | Fwd: 5’-AGATATAGGGTTTTATAATGTTGTTTAGTT-3’ | 227 | 56 | 2.5 | 0.4 | 40 |
|  | Rev: 5’-biotin-ATTTCATAAAAAAAACCAACCACTATC-3’ |  |  |  |  |  |
| MFSD1_S | Fwd: 5’-ATTTTGTTATTGTGAAGTAATTAT-3’ |  |  |  |  |  |
| LTA_1^st^ round | Fwd: 5’-ATTGATTGTTTTTTTTTTGGAATTTTAGG-3’ | 319 | 56 | 2.5 | 0.4 | 40 |
|  | Rev: 5’-ATAAAACAAAAAAACCTCACCTACTAT-3’ |  |  |  |  |  |
| LTA_nested | Fwd: 5’-ATTGATTGTTTTTTTTTTGGAATTTTAGG-3’ | 302 | 56 | 2.5 | 0.4 | 40 |
|  | Rev: 5’-biotin-CACCTACTATACRAAACCCCTAAACC-3’ |  |  |  |  |  |
| LTA_S | Fwd: 5’-TTTAAGTTTTGGGGGT-3’ |  |  |  |  |  |
| UBASH3A_1^st^ round | Fwd: 5’-AGGGGGTTAGTAGGATTTAGGA-3’ | 283 | 56 | 2.5 | 0.4 | 40 |
|  | Rev: 5’-GGTTAGTAGGATTTAGGAGGGAATTTA-3’ |  |  |  |  |  |
| UBASH3A_nested | Fwd: 5’-AGGGGGTTAGTAGGATTTAGGA-3’ | 279 | 56 | 2.5 | 0.4 | 40 |
|  | Rev: 5’-biotin-ACCTCAAACTCTTTTTAATCTTTCTAAC-3’ |  |  |  |  |  |
| UBASH3A_S | Fwd: 5’-GTTGATTTYGTGTAGGGGTT-3’ |  |  |  |  |  |
| CD3G_1^st^ round | Fwd: 5’-GGAGGTTTAGTTTTAAGTATTTGAGAGT-3’ | 212 | 56 | 2.5 | 0.4 | 40 |
|  | Rev: 5’-AATACTAAATTCCCACTCAAAAACTCAT-3’ |  |  |  |  |  |
| CD3G_nested | Fwd: 5’-GGAGGTTTAGTTTTAAGTATTTGAGAGT-3’ | 203 | 56 | 2.5 | 0.4 | 40 |
|  | Rev: 5’-biotin-TTCCCACTCAAAAACTCATCTTACAC-3’ |  |  |  |  |  |
| CD3G_S | Fwd: 5’-GGGATATTTATTTTTTTTATGAAGA-3’ |  |  |  |  |  |
| IL24_1^st^ round | Fwd: 5’-AAGTGATGGGTGATTAGGTAATGAA-3’ | 329 | 56 | 2.5 | 0.4 | 40 |
|  | Rev: 5’-GATGGGTGATTAGGTAATGAAGTA-3’ |  |  |  |  |  |
| IL24_nested | Fwd: 5’-AAGTGATGGGTGATTAGGTAATGAA-3’ | 325 | 56 | 2.5 | 0.4 | 40 |
|  | Rev: 5’-biotin-ACCAAAAATCCCCCATACTCA-3’ |  |  |  |  |  |
| IL24_S | Fwd: 5’-TGTAAATTTTTATTTTTGAAATGAT-3’ |  |  |  |  |  |
| EPHB3_1^st^ round | Fwd: 5’-TTGTTAGGTAAATATTTTTAGGGGAGTAG-3’ | 93 | 56 | 2.5 | 0.4 | 40 |
|  | Rev: 5’-TGTTAGGTAAATATTTTTAGGGGAGTAGAT-3’ |  |  |  |  |  |
| EPHB3_nested | Fwd: 5’-TTGTTAGGTAAATATTTTTAGGGGAGTAG-3’ | 92 | 56 | 2.5 | 0.4 | 40 |
|  | Rev: 5’-biotin-AACCACAAACCTACCCAACTCA-3’ |  |  |  |  |  |
| EPHB3_S | Fwd: 5’-GTAGATTTATTGTTGGGTTATT-3’ |  |  |  |  |  |
| ITPKB_1^st^ round | Fwd: 5’-GGTGTGGTTTAGTGGATGTAATT-3’ | 147 | 56 | 2.5 | 0.4 | 40 |
|  | Rev: 5’-GAATAGTTTTAATGAGGGAGTTAGGTAG-3’ |  |  |  |  |  |
| ITPKB_nested | Fwd: 5’-GGTGTGGTTTAGTGGATGTAATT-3’ | 89 | 56 | 2.5 | 0.4 | 40 |
|  | Rev: 5’-biotin-ACTCACCCACAACTATAAAAATTAAC-3’ |  |  |  |  |  |
| ITPKB_S | Fwd: 5’-AGGGAGTTAGGTAGT-3’ |  |  |  |  |  |
| CD8B_1^st^ round | Fwd: 5’-GAGTGGAGGGTGAGGATTAT-3’ | 183 | 56 | 2.5 | 0.4 | 40 |
|  | Rev: 5’-GTTATTTTTTTTGATTTTTTTGGGAAAGAG-3’ |  |  |  |  |  |
| CD8B_nested | Fwd: 5’-GAGTGGAGGGTGAGGATTAT-3’ | 133 | 56 | 2.5 | 0.4 | 40 |
|  | Rev: 5’-biotin-AAGATAGAGGTTTGTGGTTGG-3’ |  |  |  |  |  |
| CD8B_S | Fwd: 5’-ATTTGATTTTATTTGAATTTTTAAG-3’ |  |  |  |  |  |
