# Supplementary material for: DNA Methylation Signatures Triggered by Prenatal Maternal Stress Exposure to a Natural Disaster: Project Ice Storm
Source: PLoS One. 2014 Sep 19;9(9):e107653. doi: 10.1371/journal.pone.0107653 (PMC4169571; doi:10.1371/journal.pone.0107653)
Supplement: File S1 — Supporting data. (DOCX) [file pone.0107653.s010.docx]

**Supporting data**

**Methods and Materials:**

# Participants

Project Ice Storm [[1](#_ENREF_1)] includes mothers who were pregnant during the January 1998 Quebec ice storm or who became pregnant within three months of the crisis, and their children. All women were living in the Montérégie region southeast of Montreal at the time of the storm, were native French speakers, ethnically Caucasian, and were aged 18 years or older. The original sample of 178 had significantly higher education and income than the average for their region (see [[2](#_ENREF_2)] for a full description of the recruitment of the initial sample). Approximately 100 families remain active in the study. The children are equally divided among males and females.

Thirty-six youth (20 males; 16 females) provided blood samples for epigenetic analyses in October 2011 when they were on average, 13.3 years of age (SD=0.3). Youth who participated in the blood draw were compared to the sample that participated in a full psychometric assessment at 11½ years but who refused the blood draw. There were no group differences in ice storm-related maternal stress (see below), maternal psychological characteristics (state anxiety, major life events, IQ, and familial socio-economic status), or birth outcomes (gestational age, birth weight and length, head circumference, ponderal index).

# Measures and Procedures

This study was approved by the Research Ethics Board of the Douglas Hospital Research Center. We obtained informed consent from parents and informed assent from children at every assessment.

In June 1998, storm-related prenatal maternal stress was assessed using two questionnaires. A 32-point questionnaire of objective hardship (Storm32), which addressed loss (e.g. damage to residence), scope (e.g. number of days without electricity), threat (injury to self), and change (e.g. time in a shelter) (see [[3](#_ENREF_3)] for a full description of the questionnaire). Women also completed a validated French version [[4](#_ENREF_4)] of the Impact of Events Scale-Revised [[5](#_ENREF_5)] which addressed the women’s subjective distress due to the storm expressed as post-traumatic stress-like symptoms. We also collected demographic and health data from the women at that time. We have conducted assessments of children’s physical, cognitive, and behavioral outcomes since 1998.

Objective hardship assessed the severity of storm-related events experienced by the pregnant women by tapping into four categories of exposure used in other disaster studies: Threat, Loss, Scope, and Change [[6](#_ENREF_6)]. Because each natural disaster presents unique experiences, questions pertaining to each category must be tailor-made. Our scale included questions specific to the ice storm, such as days without electricity, danger due to falling ice or tree branches, and spending time in temporary shelters (see [[3](#_ENREF_3)] for the complete list of questions). Each dimension was scored on a scale of 0 – 8, ranging from no exposure to high exposure. A total objective hardship score (Storm32) was calculated by summing scores from all four dimensions using McFarlane's approach [[7](#_ENREF_7)]. In the present study, scores ranged from 5 - 21 out of a possible 32 points.

Subjective distress was assessed using a validated French version of the Impact of Event Scale – Revised (IES-R) [[4](#_ENREF_4)] This 22-item scale, widely used for assessing distress following trauma exposure, describes symptoms from three categories relevant to post-traumatic stress disorder: Intrusive Thoughts, Hyperarousal, and Avoidance. Participants responded on a 5-point Likert scale, from “*Not at all”* to “*Extremely*,” the extent to which each behavior described how they felt over the preceding seven days. Items were written to reflect symptoms relative to the ice storm. The total score was used in analyses.

To minimize the distance families had to travel blood draw clinics were held in four colleges in the Montérégie region of Québec between October 15 and November 12, 2011. The blood draw clinics were held to obtain blood samples for immune, metabolic, and epigenetic assays. The families were asked to arrive at college on the date of their choice between 07:00 and 10:00 hours since a fasting blood sample was required for a glucose tolerance assessment. A licensed phlebotomist, using an evacuated tube system with a multiple sample sleeve for venipuncture on either the left or right arm, drew a total of six blood samples at two time points: four upon arrival and two 30 minutes later, totalling 50 mL of blood. The families were provided with breakfast following the completion of the blood draw. The youths received a $50 gift card for their participation.

# T cell isolation and DNA extraction

Ten milliliters of blood was perfused in PAX gene Blood DNA tubes (Qiagen #7115, CA) and stored at 4°C before further study. The peripheral blood mononuclear cells (PBMCs) layer was isolated by centrifugation with Ficoll-Paque (GE Healthcare) and washed twice with HBSS (GIBCO, Invitrogen). T cells were isolated from PBMCs by immunomagnetic separation with Dynabeads CD3 (Dynal, Invitrogen). Beads were washed twice with PBS/FBS and incubated with the PBMCs on a nutator for 45 min at 4°C. Coated CD3+ T cells with the Dynabeads were separated using a strong magnet (DynaMag™-15 Magnet, Invitrogen) and washed three times with PBS/FBS. The cells pellet was stored at -80°C until further analysis. DNA extraction from T cells and PBMCs was performed using Wizard Genomic DNA Purification kit (Promega) according to the manufacturer’s instructions. DNA yield was measured using Qubit 2.0 Fluorometer (Invitrogen). DNA was stored at -80°C until analysis.

# Saliva collection and DNA extraction

Saliva was collected using Oragene DNA self-collection kit (OG-500) (DNA Genotek). The saliva for DNA analyses for 34 children was collected when they were 8 years of age. Saliva samples for DNA analyses of 2 children were not obtained. The saliva samples were stored at room temperature until further analysis. DNA extraction was performed using PrepIT-L2P kit (DNA Genotek) according to the manufacturer’s instructions. DNA yield was measured using NanoDrop 8000 Spectrophotometer V2.1 (Thermo Scientific). DNA was stored at -80°C until analysis.

# Infinium Human Methylation 450 BeadChip Array

We evaluated the effect of maternal exposure to disaster on DNA methylation from the T cells of 34 youth. DNA methylation from 2 youth was not obtained due to very low T cell DNA concentrations. Infinium Human Methylation 450 BeadChip can measure methylation levels of more than 480 000 CGs and investigate DNA methylation profiling on a genome-wide scale. Infinium Human Methylation 450 was completed by Genome Quebec.

Microarray quality was assessed using plots created using minfi Bioconductor [[8](#_ENREF_8)]. This package was also used to background correct and normalize microarrays to control probes. Probe intensities were then transformed to beta-values (M/(M+U)), where M and U denote the methylated and unmethylated probe intensities, respectively. Although beta-values can be conveniently interpreted as methylation levels, association analyses were carried out using logit-transformed beta values (M-values) [[9](#_ENREF_9)]. Significant associations with variables of interest were identified using the minfi’s “dmpFinder” function with default settings. In order to reduce the number of association tests, only probes with sufficiently high variance across the samples were tested. These probes had an inter-quartile range corresponding to a 10% change in methylation across the samples. Furthermore, since samples were obtained from both males and females, probes for chromosomes X and Y were excluded. The remaining 10553 probes were tested for association with the object hardship, “Storm32”, and subjective distress “IES-R”. False discovery rates (FDR) were calculated from the p-values of these tests in order to control for multiple testing using the Benjamini-Hochberg algorithm. A probe was called significantly associated if its false discovery rate was at most 0.2.

Potential interactions between the measure of objective hardship, Storm32, and gender within the microarray data were investigated using the limma R package [[10](#_ENREF_10)] (interactions are not supported by the minfi “dmpFinder” function). As above, tests were applied only to the 10553 probes with sufficient variance located on an autosome, and errors due to multiple testing were controlled by restricting the false discovery rate to 0.2. None of the tests identified any interactions between objective hardship and gender although there were a few cases where a probe was significantly associated with objective hardship in one gender only.

Genomic regions were tested for enrichment with positive or negative associations with objective hardship using Fisher’s exact test. To apply the test, each probe (i.e. profiled CG) was linked to a nearest gene transcription start site order to identify a set of “profiled” genes. A gene was said to be “significantly associated” with objective hardship if it was linked to a significantly associated probe/CG. Each genomic region was tested for enrichment with significantly associated genes relative to the set of all profiled genes using Fisher’s exact test.

Heatmaps illustrate Beta-value variation for micrroary probes (rows) across study participants (columns). Samples and probes are clustered using the Ward algorithm with Pearson’s correlation as the distance metric. Relatively lower methylation levels are shown in green and higher methylation levels in red.

# Bisulfite treatment and pyrosequencing

The bisulfite treatment of 250ng genomic DNA was performed using the EZ DNA Methylation-Gold Kit (zymo Research) according to the manufacturer’s protocol. Bisulfite-converted DNA was subjected to PCR amplification of the specific CGs. For each gene, 2 pairs of primers were used for amplification of two rounds of PCR. The nested reverse primers were biotin labelled for pyrosequencing. The numbers of CpG sites that are covered by both rounds, the Illumina target ID and probe ID are provided in ***Table S1***.

EpiTaq HS Polymerase (TaKaRa) was used for bisulfite-treated DNA amplification. The primer sequences and PCR amplification conditions are shown in ***Table S2***. The PCR products were separated by gel electrophoresis and visualized under UV light to confirm product size. 20ul of specific PCR products were subjected to pyrosequencing using PyroMarkQ24 (Qiagen) according to the manufacturer’s protocol. Biotin labelled DNA was purified following the manufacture’s protocol. Briefly, biotin labelled DNA was immobilized on streptavidin-sepharose beads (GE Healthcare). After washing and denaturation, the biotinylated single stranded DNA was released into annealing buffer containing the sequencing primer.

Two positive methylation controls (50% and 100% methylation) using M.SssI enzyme (New England biolabs) treated DNA and negative controls using WGA REPLI-g Mini Kit (Qiagen) amplified DNA were served in Pyrosequencing. Positive and negative controls were performed in each run. The methylation percentage of each CG was subsequently analyzed using the PyroMark Q24 software (Biotage).

# SCG5 promoter cloning

Two fragments of SCG5 promoter were cloned by PCR amplification from the HEK cell cDNA at positions 32933343-32933992 (chromosome 15) using primers tttAGATCTACTTGATGGTTTGTGTGGACG and tttCCATGGAAACTGTACTCACCGAGGGG and positions 32933343-32934034 (chromosome 15) using primers tttAGATCTACTTGATGGTTTGTGTGGACG and tttCCATGGTACCAAATCTCGGGTTGGCG. The two corresponding anti-sense fragments of SCG5 promoter were cloned by PCR at positions 32933343-32933992 (chromosome 15) using primers tttCCATGGACTTGATGGTTTGTGTGGACG and tttAGATCTAAACTGTACTCACCGAGGGG and positions 32933343-32934034 (chromosome 15) using primers tttCCATGGACTTGATGGTTTGTGTGGACG and tttAGATCTTACCAAATCTCGGGTTGGCG. PCR products were digested with *Bgl*II and *Nco*I (New England Biolabs, Whitby, Canada) and cloned into the pCpGL-reporter containing the luciferase reporter gene [[11](#_ENREF_11)]. All clones were sequence-verified using pCpGL-sequencing forward primer: 5’- gtgagcaaacagcagattaaaagga-3’ and reverse primer: 5’- gggaccagggcatacctctt-3’.

# In vitro pCpGL-SCG5 promoter methylation

Plasmid constructs were methylated *in vitro* using CpG methyltransferase M.*Sss*I (New England Biolabs). Brieﬂy, 10 µg plasmid, 1 µl SAM (32 mM) and 2 µL SssI methylase (4U/µl) in a 50 µl reaction were incubated at 37°C for overnight and subsequently inactivated (65°C, 20min). Plasmids were puriﬁed using QuickClean II PCR Extraction Kit (GenScript, Piscataway, USA). Methylation-speciﬁc restriction digestion *Hpa*II and *Msp*I (New England Biolabs) were used to confirm the complete methylation.

# Cell line and transfection

Human embryonic kidney 293 cells (HEK293) (ATCC) were grown in DMEM high glucose (Gibco, Invitrogen, Life Technologies, Carlsbad, CA) in controlled environment (5% CO_2_, 37°C). 50,000 cells were platted on 6 well plates 24h before transfection. 300ng of vector was transiently transfected using calcium phosphate method based on [[12](#_ENREF_12)]. Cell media was replaced 16h after transfection and the cells were harvested 48 hour post transfection.

# Luciferase activity assay

Cells were washed with PBS (Invitrogen, Life Technologies, Carlsbad, CA) and harvested using a cell scraper (Sarstedt, Germany). Cells were spun down (5min at 1000rpm) and lysed in 30µl lysis buffer (25mM Tris-Phosphate (pH=7.8), 10% Glycerol, 1% Triton-X, 1mg/ml BSA, 2.5mM EDTA (pH=8.0) and 1x Complete-mini EDTA free protein inhibitors (Roch, Germany)) for 5 min on ice. The lysate was spun for 5 min at 13,000 rpm and the supernatant was removed for activity evaluation. 10µl of the lysate was added to each well with 100µl Luciferase assay substrate (Promega, USA). The reactions were read using Lumat LB9507 (Berthold Technologies, Germany) and normalized to the total protein concentration per sample.

The protein concentration was measured using Bradford reaction assay (Biorad, USA) according to the manufacturer recommendations, and read using DU730 UV/Vis spectrophotometer (Beckman Coulter, USA).

# Ingenuity pathway analysis (IPA)

The genes containing CGs which showed differential DNA methylation in T cells were uploaded and classified by IPA software (www.ingenuity.com) which identified the most significant biological functions and/or diseases based on a large number of manually collected relationships between genes from the scientific literature. A right-tailed Fisher’s exact test was used to calculate the Gene enrichment. Biological functions with a cut off p-value less than 0.05 were considered statistically significant.

# Statistical Analysis

The Illumina 450K BeadChip statistical analyses were performed using R packages and are described earlier. All other analyses were performed using SPSS (Version 20, SPSS Inc., Chicago IL, USA). Associations were calculated using Pearson’s correlation coefficient which was corrected according to Bonferroni. All p-values reported are two-sided.

**Figure legends:**

# Figure S1

**The correlation between objective hardship score (Storm32) and methylation data from Illumina Human Methylation 450K BeadChip Array in 12 CGs associated with 9 genes.**

X-axis indicates the percentage methylation of CGs from pyrosequencing and y-axis indicates the beta-value from 450K BeadChip. Blue squares indicate male and green diamonds indicates female. Dashed blue line represents the fitting line in males and green in females.

# Figure S2

**The correlation between objective hardship score (Storm32) and methylation data from pyrosequencing.**

Correlations between objective hardship score (Storm32) and methylation level of CG(s) in (A)*MFSD1*, (B)*CD3G*, (C)*UBASH3A*, (D)*IL24*, (E)*EPHB3*, (F)*ITPKB* and (G)*CD8B*. Blue squares indicate male and green diamonds indicates female. Dashed blue line represents the fitting line in males and green in females. Track on the screenshot of Integrative Genomics Viewer (IGV) window marks the location of the CGs examined using pyrosequencing.

# Figure S3

**CTLA4 Signaling in Cytotoxic T Lymphocytes.**

Genes whose methylation levels are positively correlated with objective hardship are colored in red and those whose methylation levels are negatively correlated with objective hardship are colored in blue. CD247: CD247 molecule; FYN: a membrane-associated tyrosine kinase; CD3E: CD3-epsilon polypeptide; HLA-DMB: Major Histocompatibility Complex, Class II, DM Beta; CD3D: CD3d Molecule, Delta; CTLA4: cytotoxic T-lymphocyte-associated protein 4; CD3G: CD3-gamma polypeptide; CD28: CD28 Molecule; LCK: lymphocyte-specific protein tyrosine kinase; SYK: spleen tyrosine kinase; ZAP70: zeta-chain (TCR) associated protein kinase 70kDa; HLA-DOB: Major Histocompatibility Complex, Class II, DO Beta; PIK3CD: phosphatidylinositol-4,5-bisphosphate 3-kinase, catalytic subunit delta; PIK3R2: phosphoinositide-3-kinase, regulatory subunit 2 (beta); LCP2: Lymphocyte Cytosolic Protein 2; PPP2R5C: protein phosphatase 2, regulatory subunit B', gamma; PPP2R5E: protein phosphatase 2, regulatory subunit B', epsilon isoform.

# Figure S4

**T Cell Receptor Signaling.**

Genes whose methylation levels are positively correlated with objective hardship are colored in red and those whose methylation levels are negatively correlated with objective hardship are colored in blue. CD247: CD247 molecule; FYN: a membrane-associated tyrosine kinase; CD3E: CD3-epsilon polypeptide; CSK: C-Src Tyrosine Kinase; PLCG1: Phospholipase C, Gamma 1; NFATC1: Nuclear Factor Of Activated T-Cells, Cytoplasmic, Calcineurin-Dependent 1; HLA-DMB: Major Histocompatibility Complex, Class II, DM Beta; CD3D: CD3d Molecule, Delta; CTLA4: cytotoxic T-lymphocyte-associated protein 4; CD8B: CD8b molecule; CD3G: CD3-gamma polypeptide; CD28: CD28 Molecule; LCK: lymphocyte-specific protein tyrosine kinase; ACTR3: ARP3 Actin-Related Protein 3 Homolog (Yeast); NFKBIA: nuclear factor of kappa light polypeptide gene enhancer in B-cells inhibitor, alpha; BCL10: B-Cell CLL/Lymphoma 10; ZAP70: zeta-chain (TCR) associated protein kinase 70kDa; PIK3CD: phosphatidylinositol-4,5-bisphosphate 3-kinase, catalytic subunit delta; PIK3R2: phosphoinositide-3-kinase, regulatory subunit 2 (beta); LCP2: Lymphocyte Cytosolic Protein 2; ITK: IL2-inducible T-cell kinase. PAG1: phosphoprotein associated with glycosphingolipid microdomains 1.

**Table S1.**

**The numbers of CGs and analyzed sequences using pyrosequencing.**

**Table S2.**

**Forward, reverse, pyrosequencing primer sequences and PCR conditions used for bisulphite sequencing.**

**Table S3.**

**1675 CGs significantly correlated with objective hardship levels (Storm32).**

**Table S4.**

**Selected CGs/genes for pyrosequencing.**

**Table S5.**

**Pathway information.**

**Additional references:**

1. King S, Dancause K, Turcotte-Tremblay A-M, Veru F, Laplante DP (2012) Using Natural Disasters to Study the Effects of Prenatal Maternal Stress on Child Health and Development. Birth Defects Research Part C: Embryo Today: Reviews 96: 273-288.

2. Laplante DP, Barr RG, Brunet A, Galbaud du Fort G, Meaney MJ, et al. (2004) Stress during pregnancy affects general intellectual and language functioning in human toddlers. Pediatric Research 56: 400-410.

3. Laplante DP, Zelazo PR, Brunet A, King S (2007) Functional play at 2 years of age: Effects of prenatal maternal stress. Infancy 12: 69-93.

4. Brunet A, St-Hilaire A, Jehel L, King S (2003) Validation of a French version of the Impact of Event Scale - Revised. Canadian Journal of Psychiatry 48: 55-60.

5. Weiss DS, Marmar CR (1997) The Impact of Event Scale - Revised. In: Wilson JP, Keane TM, editors. Assessing psychological trauma and PTSD: A practitioner's handbook. New York: Guilford. pp. 399-411.

6. Bromet E, Dew MA (1995) Review of Psychiatric Epidemiologic Research on Disasters. Epidemiologic Reviews 17: 113-119.

7. McFarlane AC (1988) Relationship between psychiatric impairment and a natural disaster: The role of distress. Psychological Medicine 18: 129-139.

8. Hansen KD, and M. Aryee. (2012) "Minfi: Analyze Illumina’s 450k Methylation Arrays." R package version 1.2

9. Du P, Zhang X, Huang CC, Jafari N, Kibbe WA, et al. (2010) Comparison of Beta-value and M-value methods for quantifying methylation levels by microarray analysis. BMC Bioinformatics 11: 587.

10. Smyth GK (2005) Solutions using R and Bioconductor. In: Carey V, Gentleman R, Dudoit S, Irizarry R, Huber W, editors. Bioinformatics and Computational Biology Vol 1 Springer. pp. 397-420.

11. Klug M, Rehli M (2006) Functional analysis of promoter CpG methylation using a CpG-free luciferase reporter vector. Epigenetics 1: 127-130.

12. Rouleau J, Tanigawa G, Szyf M (1992) The mouse DNA methyltransferase 5'-region. A unique housekeeping gene promoter. J Biol Chem 267: 7368-7377.
